# The Effects of Low-Vision Rehabilitation on Reading Speed and Depression in Age Related Macular Degeneration: A Meta-Analysis

**DOI:** 10.1371/journal.pone.0159254

**Published:** 2016-07-14

**Authors:** Noura Hamade, William G. Hodge, Muhammad Rakibuz-Zaman, Monali S. Malvankar-Mehta

**Affiliations:** 1 Department of Epidemiology and Biostatistics, Schulich School of Medicine and Dentistry, University of Western Ontario, London, ON, Canada; 2 Department of Ophthalmology, Schulich School of Medicine and Dentistry, University of Western Ontario, London, ON, Canada; University Medical Center Goettingen, GERMANY

## Abstract

**Importance:**

Age related macular degeneration (AMD) is a progressive eye disease that, as of 2015, has affected 11 million people in the U.S. and 1.5 million in Canada causing central vision blindness. By 2050, this number is expected to double to 22 million. Eccentric vision is the target of low-vision rehabilitation aids and programs for patients with AMD, which are thought to improve functional performance by improving reading speed and depression.

**Objective:**

This study evaluates the effect of various low-vision rehabilitation strategies on reading speed and depression in patients 55 and older with AMD.

**Data Sources:**

Computer databases including MEDLINE (OVID), EMBASE (OVID), BIOSIS Previews (Thomson-Reuters), CINAHL (EBSCO), Health Economic Evaluations Database (HEED), ISI Web of Science (Thomson-Reuters) and the Cochrane Library (Wiley) were searched from the year 2000 to January 2015.

**Study Selection:**

Included papers were research studies with a sample size of 20 eyes or greater focused on AMD in adults aged 55 or older with low vision (20/60 or lower).

**Data Extraction and Synthesis:**

Two independent reviewers screened and extracted relevant data from the included articles. Standardized mean difference (SMD) was chosen as an effect size to perform meta-analysis using STATA. Fixed- and random-effect models were developed based on heterogeneity.

**Main Outcomes:**

Reading Speed and Depression Scores.

**Results:**

A total of 9 studies (885 subjects) were included. Overall, a significant improvement in reading speed was found with a SMD of 1.01 [95% CI: 0.05 to 1.97]. Low-vision rehabilitation strategies including micro-perimetric biofeedback, microscopes teaching program significantly improved reading speed. Eccentric viewing training showed the maximum improvement in reading speed. In addition, a non-significant improvement in depression scores was found with a SMD of -0.44 [95% CI: -0.96 to 0.09].

**Conclusion:**

A considerable amount of research is required in the area of low-vision rehabilitation strategies for patients with AMD. Based on current research, low-vision rehabilitation aids improve reading speed. However, they do not have a significant effect on depression scores in those 55 and older with AMD.

## Background

AMD is a leading cause of irreversible visual loss worldwide and the leading cause of vision loss in U.S. [1] and Canada [2]. Currently, more than 11 million people in the U.S. [1] and 1.5 million in Canada [2] are living with AMD. By 2050, this number is expected to double to 22 million people with AMD in the US. [1] An estimated 8.7% of the world’s population has AMD [3]. There are almost 200,000 new cases of AMD every year in Canada, which does not only create a great economic burden for the country but also has devastating consequences on patients' lives [2].

The global cost of visual impairment due to all causes, in the US, is $3 trillion for 733 million people living with low-vision and blindness, of which $255 billion is due to AMD [1]. In U.S., Canada, and Cuba, collectively, direct cost of visual impairment due to AMD is US$98 billion [1]. According to a study by Brown et al., not only is AMD associated with 40% decrease in quality of life but also has a $2.6 billion impact on Canada’s gross domestic product [4]. A large percentage of that economic burden comes from productivity loss due to person’s inability to function independently because of their low vision. This also leads to people with AMD earning 38% less than a person with no disability [2]. In addition those with AMD, have much lower employment rates compared to those from the general unaffected population [5].

AMD is a progressive eye condition caused by macular pathology, which is located at the back of the eye and responsible for the central sharp 20/20 vision. AMD causes distortion of vision, scotomas and, as it progresses, central vision loss. On the other hand, peripheral vision is mostly spared and is usually the target of vision aids [6]. Even with vitamin supplements and intravitreal anti-VEGF injections, AMD is still, as of now, a mostly incurable disease with causes not well understood. Therefore, management and rehabilitation through vision aids and low-vision strategies is important for the continued independence of those living with AMD [7].

Various low-vision rehabilitation aids exist to improve reading speed [8–13] and depression scores [14–16]. Reading is affected most severely as vision progressively degenerates over time [17]. Reading speed is a good measure of functional and reading ability and thus, reading speed was chosen as the primary outcome of this meta-analysis. Additionally, any improvements in reading speed after low-vision rehabilitation may be informative about the improvements in visual functioning in those with low vision from maculopathy. Although, the primary focus of low-vision rehabilitation is mainly improving visual function, it can also affect AMD patient’s psychological health [18]. AMD, as mentioned earlier, has a great impact on psychological well-being because of its devastating effects on patients’ independence in their daily lives. This has been found to lead to severe depression among patients with AMD causing decreased quality of life [19]. It is important to recognize depression in patient with AMD to allow for addressing this condition to improve the patients’ standard of living.

We conducted a systematic review and meta-analysis to assess the effectiveness of low-vision rehabilitation strategies used by those aged 55 and older with AMD in improving patients’ function through reading speed and depression scores.

## Methods

### Studies and Participants

Studies were included if they were a research study, (excluding editorials, opinions and case reports), randomized control trial or observational study. Additionally, studies considering 20 or more eyes of patients with AMD, both dry and wet, along with low-vision aids and devices (and rehabilitation training) were included. Studies had to consider specifically low-vision patients using the low-vision definition by the CNIB, which defines low-vision as a visual acuity score of 20/60 or lower, in order to be included. Studies considering patients aged 55 and older with AMD were included. There were no limits on either the length of follow-up or time since rehabilitation. Only human studies, published in English were included in this systematic review. Due to advancements in technology, low-vision rehabilitation techniques may change over a period and thus, to be consistent, year 2000 was selected as a cut-off year. Studies that satisfied the above criteria and included at least one of the main outcomes of interest—reading speed and depression scores—were included in the review.

### Search Strategy

We adhered to the Preferred Items for Systematic Reviews and Meta-Analyses (PRISMA) guidelines (S1 File) [20]. Computer databases including MEDLINE (OVID), EMBASE (OVID), BIOSIS Previews (Thomson-Reuters), CINAHL (EBSCO), Health Economic Evaluations Database (HEED), ISI Web of Science (Thomson-Reuters) and the Cochrane Library (Wiley) were searched from the year 2000 to February 2015. The reference lists of all the included articles were hand searched to find potentially relevant studies. Search strategies were constructed utilizing database specific subject headings and keywords for “Low-vision rehabilitation” and “AMD”. Each strategy was modified to complement the specific database and platform. The general search strategy included AMD (“retina macula age related degeneration”, “age related macular degeneration” and “retina macula degeneration”), low-vision (“visual disorder”, “low-vision” and “visual impairment”), and vision aids (“spectacles”, “reading”, “visual aid”, “low-vision aid”, “rehabilitation” and “therapy”). The detailed search strategy for MEDLINE, EMBASE and CINAHL has been provided in S2 File.

Grey literature was identified by searching the conference abstracts of the Canadian Ophthalmology Society meeting (COS), American Academy of Ophthalmology annual meeting (AAO), European Society of Ophthalmology (SOE), and the Association for Research in Vision and Ophthalmology annual meeting (ARVO). The Networked Digital Library of Thesis and Dissertations (NDLTD) and the Canadian Health Research Collection (Ebrary) were also searched for relevant content. Google was used to search for additional web-based materials and information. OVID AutoAlerts were set up to send monthly updates with any new literature. All of the data was accessed online, no human subjects were recruited for the study, and therefore, ethics approval was deemed unnecessary.

### Study Selection

Selection was based on the inclusion criteria and narrowed down by three levels of screening. After importing all the studies into EPPI Reviewer 4.0 (by EPPI-Centre, Social Science Research Unit, the Institute of Education, the University of London, UK), an automatic duplication check was done by EPPI followed by two manual duplication checks done by a reviewer (NH) to remove all duplicates. After duplicate removal, Level 1 screening (title screening) was performed where studies that did not look at AMD or low-vision rehabilitation strategies were excluded. Following that, Level 2 screening (abstract screening) was performed on the included records to exclude all titles that were editorials, opinions or case reports. Level 3 screening (full text) was performed to include all studies that had a visual acuity of at least 20/60 (logMAR of 0.47), sample size of 20 or more eyes, and subjects aged 55 and older. See S3 File for detailed screening questions. At each level of screening, two reviewers (NH and MRZ) independently screened the studies and agreements and disagreements were calculated using Cohen’s kappa (κ) coefficient. Disagreements were resolved by consensus and if consensus was not reached then a third reviewer intervened to resolve the disagreements.

### Data Collection Process

Data extraction, from the included 9 articles, was done separately for each reading speed and depression scores with both baseline and follow up information. A different Table was developed to present summary information including author, year of publication, study design, study location and the number of participants in each of the included studies (Table 1). Basic demographic characteristics such as mean age, standard deviation (SD), gender, and visual acuity were collected. When extracting data, the Cochrane handbook was used to obtain SD from range, median or p-value when present [21]. Visual acuity data was converted to logMAR unit.

**Table 1:** Study Information and Patient Baseline Characteristics.

Author	Year	Study Design	Study Location	Group	N	Age (Mean)	Age (SD)	Baseline Visual Acuity (Mean)	Baseline Visual Acuity (SD)	Cases with AMD (%)
Smith et al. [10]	2005	Randomized Control Trial	England	Case	80	81	2.0	0.82	0.125	100
				Control	82			1.00	0.085	
Vingolo et al. [13]	2007	Prospective Cohort	Italy		27	74.5	5.25	0.09	0.03	100
Seiple et al. [11]	2011	Randomized, repeated measure crossover	U.S.	Case	30	76	8.5	0.8	0.225	100
				Control	6	78.4	8.8	0.9	0.225	
Scanlan et al. [12]	2004	Case control	Canada	Case	32	81	6.0	0.89	0.208	100
				Control	32					
Nilsson et al. [9]	2003	Prospective cohort	Sweden		20	77.4	6.0	0.042	0.016	100
Cheong et al. [8]	2009	Cross sectional	China		29	80	6.0	0.81	0.3	100
Brody et al. [15]	2006	Randomized control trial	U.S.	Case	12	81.5	7.5	1.26	0.45	100
				Control	20					
Horowitz et al. [16]	2006	Prospective cohort	U.S.		438	80.4	7.43	n/a	n/a	69.7
Girdler et al. [14]	2010	Randomized Control trail	Australia	Case	36	79.4	7.2	0.97	0.5	79.2
				Control	41	80.4	6.7	1.0	0.46	

*n/a: Information was not presented in the study and could not be calculated

### Statistical Analysis

The statistical analysis was done using STATA v. 13.0 (STATA Corporation, College Station, TX). All analysis results were presented in forest plots and expressed in standard mean differences (SMD), because of the continuous nature of the outcomes of interest, using 95% confidence intervals (CI). To compute SMD for each study, the mean pre- and post-operative values for each outcome measure was divided by the SD for that same outcome measure. Mean measures of reading speed that were collected before and after low-vision rehabilitation intervention were used to compute the forest plot for reading speed. Similarly, the depression scores before and after rehabilitation intervention were used to compute the forest plot for depression scores.

To test heterogeneity, *I*^2^ statistics [22], *Z*-value, and χ^2^ statistics [23] were computed. An *I*^2^ value of less than 50% or a low heterogeneity case resulted in fixed-effect computations and an *I*^2^statistics of 50% or more or a high heterogeneity case resulted in random-effect computations. Additionally, a high *Z*-value, a low p-value (< 0.01) and a large *χ*^2^ value implied significant heterogeneity and therefore, a random-effect model using DerSimonian and Laird methods was computed. Funnel plots were used to present publication bias in included studies where asymmetry indicated the presence of publication bias.

### Risk of Bias in Individual Studies

The GRADE guidelines were used for bias assessment in the included studies [24]. The risk of bias was based on the study design, whether randomized control trial or observational. The final grade varied from very high to very low based on the level of completeness of the requirements of study level listed above.

## Results

### Search results

**Fig 1:**
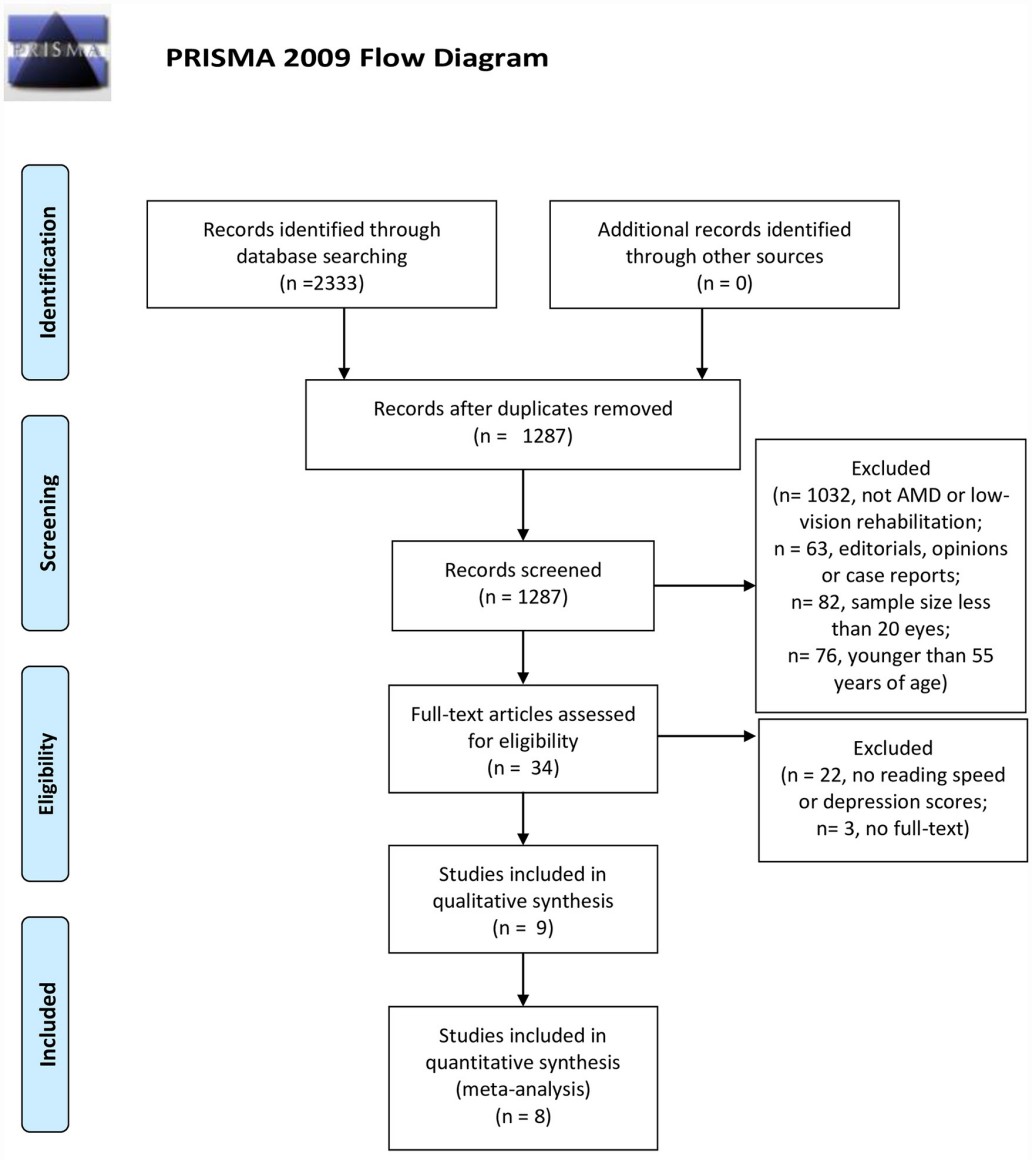
PRISMA Flow Diagram for Effects of Low-Vision Rehabilitation on Reading Speed and Depression in Age Related Macular Degeneration. *From*: Mohar D, Liberati A, Tetzlaff J, Altman DG, The PRISMA Group (2009). *P*referred *R*eporting *I*tems for *S*ystematic Reviews and *A*nalyses: The PRISMA Statement. PLOS Med 6(6): e1000097. doi:10.1371/journal.pmed1000097 For more information, visit www.prisma-statement.org.

Searches done from 2000 to February 2015 yielded 2333 articles. After duplicate removal, 1287 records remained and were screened for title (Level 1 screening) and abstract (Level 2 Screening) resulting in 192 records. Studies (1032 records) that did not look at AMD or low-vision rehabilitation strategies were excluded after title screening. Studies (63 records) that were editorials, opinions or case reports were excluded after abstract screening. Studies (82 records) that had a sample size of less than 20 eyes and studies (76 records) that had subjects younger than 55 were excluded after full-text screening. Thirty-four articles remained after full-text screening (Level 3 screening). At this stage, a final check was performed which resulted in exclusion of 22 articles due to lack of measures for reading speed or depression and three articles due to low quality score (articles with abstract only). Therefore, nine articles were included in the qualitative analysis and eight in the quantitative analysis. The PRISMA chart (Fig 1) presents a flow chart of the study selection process.

The kappa statistic for agreement between the two reviewers for Levels 1, 2, and 3 screening were 34%, 67% and 55% respectively. The reason for such a low kappa score could be due to the number of undecided or unclear response options picked while screening.

### Study Characteristics

Nine articles (885 subjects) were included. [8–16]. Table 1 summarizes the characteristics of each included study. These studies were located in various countries including three in the U.S., and one in each of England, Italy, Canada, Sweden, China and Australia. Four of the nine studies were randomized control trials, three were prospective cohorts, a case control, and a cross sectional. The mean ages were 74.5 or older in all studies. Table 2 presents the extracted information from the five studies that provided reading speed scores [9–13]. While other three studies provided depression scores (Table 3) and were used to conduct an analysis on depression scores [14–16]. One of the included article [8] provided reading speed, however, it was not used in the quantitative analysis because of an unconventional measurement for reading speed, log words per minute (log wpm), that could not be converted to match the units presented in other studies.

**Table 2:** Outcome of Reading Speed after intervention.

Author	Follow up (months)	Scale	Baseline Reading Speed for Case group (Mean wpm)	Baseline Reading Speed for Case group (SD wpm)	Baseline Reading Speed for Control group (Mean wpm)	Baseline Reading Speed for Control group (SD wpm)	Follow up Reading speed for Case group (Mean wpm)	Follow up Reading speed for Case group (SD wpm)	Follow up Reading speed for Control group (Mean wpm)	Follow up Reading speed for Control group (SD wpm)	P-value	Reading Task
Nilsson et al. [9]	5	Eccentric Viewing Training	9.0	5.8	-	-	68.3	19.4	-	-	<0.001	New Trained retinal locus (TRL): horizontally scrolled text
Smith et al. [10]	3	Spectacles	79	58	67	49	73	54	67	52	0.58	MNREAD Activity Chart
Seiple et al. [11]	4.5	Reading Rehabilitation Training	58.9	33.75	49.3	-	49.8	33.75	0.96	1.3	<0.001	MNREAD Activity Chart
Scanlan et al. [12]	3	Microscopes Teaching Program	21.55	12.72	22.13	15.56	39.0	22.6	21.0	-	0.003	Pepper Visual Skills for Reading Test
Vingolo et al. [13]	2.5	Microperimetric Biofeedback	25	21.1	-	-	45	21.1	-	-	0.031	Short printed sentences

**Table 3:** Outcome of Depression.

Author	Follow up (months)	Intervention	Outcome	BaselineDepression ScoreFor Case (Mean)	BaselineDepression ScoreFor Case (SD)	BaselineDepression ScoreFor Control (Mean)	BaselineDepression ScoreFor Control (SD)	Follow upDepression scoreFor Case(Mean)	Follow upDepression scoreFor Case(SD)	Follow upDepression scoreFor Control (Mean)	Follow upDepression scoreFor Control(SD)	P-value
Brody et al. [15]	6	Self-management program	Geriatric Depression Scale	7.5	2.19	7.8	2.23	4.58	2.42	6.80	2.96	0.001
Horowitz et al. [16]	6	Assistive device use	20-item Center for Epidemiologic Studies-Depression Scale	11.5	10.0	-	-	10.5	9.1	-	-	0.03
Girdler et al. [14]	3	Vision Management Program	Geriatric Depression Scale	8.05	0.67	11.02	0.24	7.52	0.67	10.83	0.24	< 0.01

### Effect on Reading Speed

**Fig 2:**
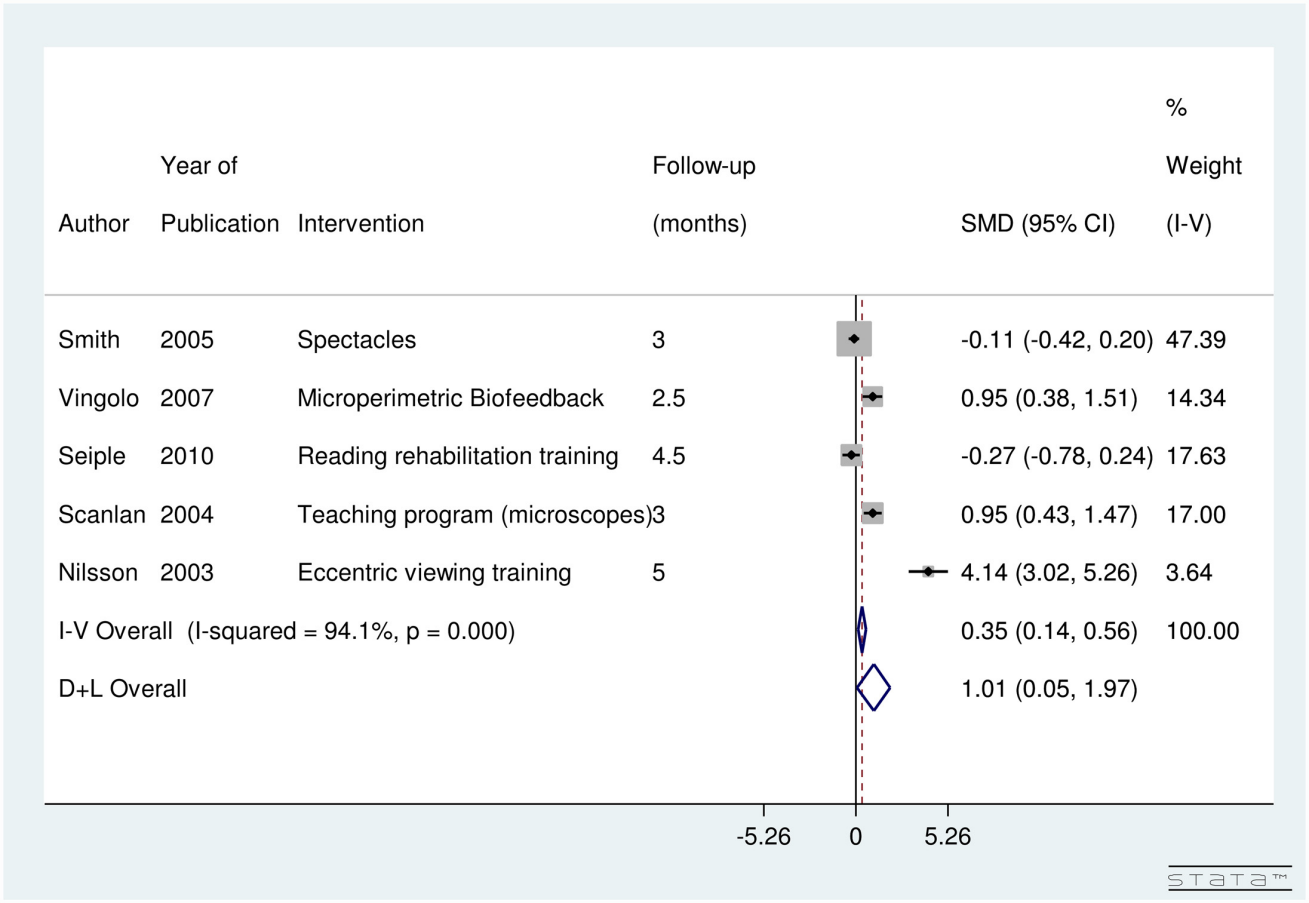
Forest Plot showing Significant Improvement in Reading Speed in Age Related Macular Degeneration Patients with various Interventions.

Five studies (309 subjects) looked into the impact of low-vision rehabilitation strategies on reading speed (wpm). The forest plot for reading speed presented in Fig 2 shows the SMD summary effect. Due to evidence of significant high heterogeneity between studies, I-squared value of 94.1% (p-value < 0.001), random-effect model was computed. Those who underwent low-vision rehabilitation showed significant improvements in reading speed compared to pre-intervention with an SMD of 1.01 [95% CI: 0.05 to 1.97] and since the summary effect does not cross the line of no-difference (thick line). Fig 2 suggests that low-vision rehabilitation strategies and devices had a significant effect on improving reading speed.

The study by Cheong et al. (29 subjects) also looked into the impact of low rehabilitation strategies on reading speed, however, it measured reading speed using log wpm which was incompatible with the rest of the included studies [8]. Therefore, it was not included in the meta-analysis. Cheong et al. showed that patients using line guides experienced a significant improvement in reading speed [8].

Looking at individual studies, low-vision rehabilitation strategies including microperimetric biofeedback [13], microscopes teaching program [12] and eccentric viewing training [11] significantly improved reading speed. Details on low-vision rehabilitation techniques are provided in Table 4. The maximum improvement in the reading speed was shown by eccentric viewing training program [9].

**Table 4:** Description of rehabilitation strategies included in the meta-analysis.

Rehabilitation Strategy	Description
Eccentric Viewing Training	Scanning laser ophthalmoscope was used to help participants locate a useful retinal focus to be trained for reading
Spectacles	Test spectacles were used for near and distance vision as bifocals or separate pairs based on participants’ preferences
Reading Rehabilitation Training	Training based on three modules: visual awareness and eccentric viewing, control of reading eye movements, and reading practice with sequential presentation of lexical information
Microscopes Teaching Program	Teaching programs included: reviewing reading techniques such as (eccentric viewing, focal distance, scrolling, and lighting), correcting poor reading skills, assigning increasingly difficult reading exercises, and answering participants' questions.
Microperimetric Biofeedback	Preferred retinal focus is recorded to be presented to the AMD patient to increase fixation stability

### Effect on Depression Scores

**Fig 3:**
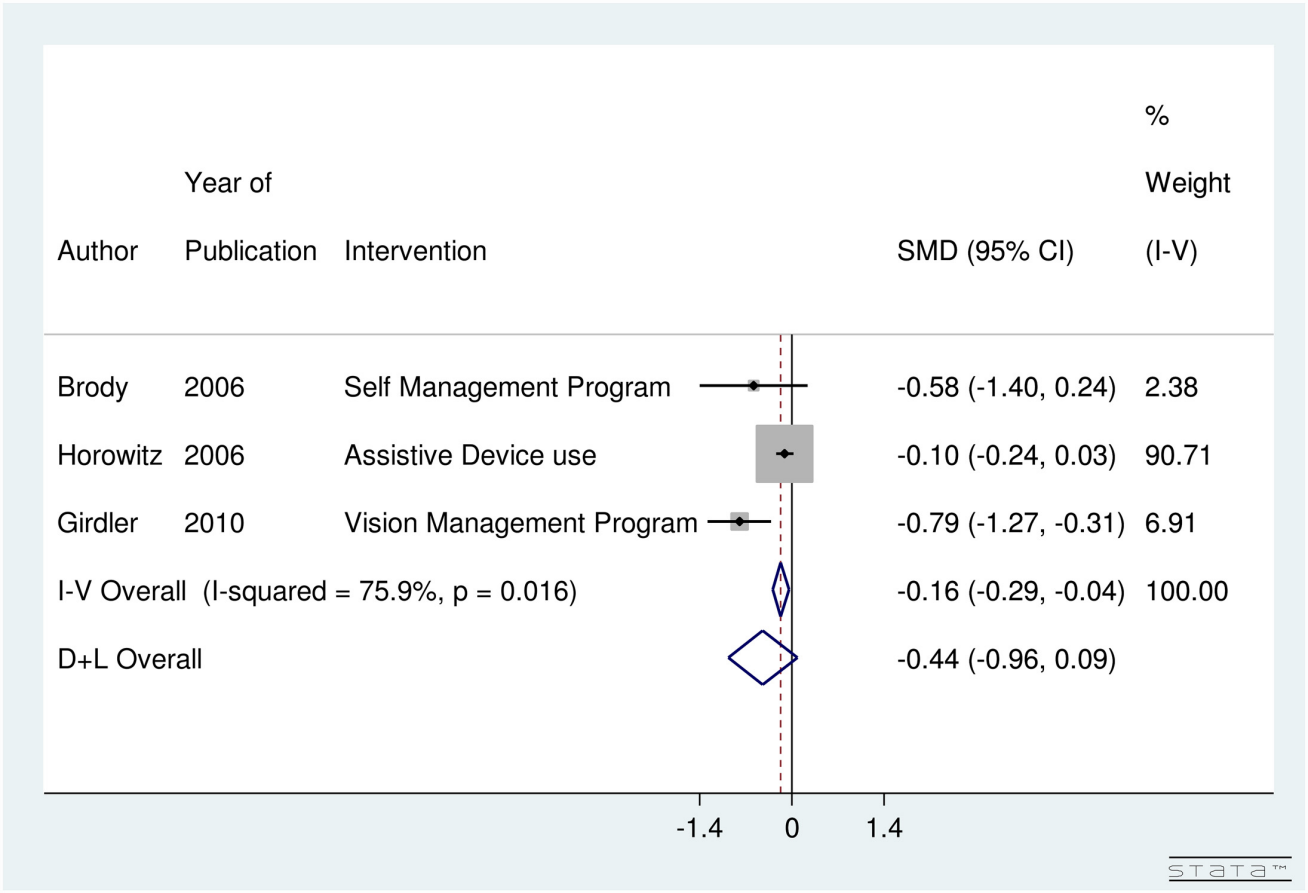
Forest Plot showing Non-Significant Improvement in Depression Score in Age Related Macular Degeneration Patients with various Interventions.

Three studies (547 subjects) looked into the impact of low-vision rehabilitation strategies on depression scores. The forest plot for depression scores presented in Fig 3 shows the SMD summary effect under the random-effect model due to evidence of significant heterogeneity, I-squared value of 75.9% (p-value < 0.016). Those who underwent low-vision rehabilitation showed a non-significant improvement in depression scores compared to pre-intervention with an SMD of -0.44 [95% CI: -0.96 to 0.09] and since the summary effect crosses the line of no-difference (thick line). Based on Fig 3, low-vision rehabilitation strategies and devices showed a non-significant effect on improving depression.

### Risk of bias

The risk of bias assessment is presented in Table 5 with the final grade for every study identified. Five [8, 12–14, 16] of the nine studies identified had a high risk of bias after evaluation. While the other four were identified with one of each very high [9], moderate [11], low [15] and very low [10] risks of bias based on the study level. However, due to the small number of identified studies in this area, all studies were included in the quantitative analysis.

**Table 5:** Risk of Bias Assessment for individual studies.

Author	Study Design	Random Sequence Generation	Allocation Concealment	Blinding	Incomplete Outcome Data Addressed	Score
Smith et al. [10]	Randomized control trial	Yes	Yes	Yes	No	Very Low
Vingolo et al. [13]	Prospective Cohort	No	No	No	Yes	High
Seiple et al. [11]	Randomized, repeated measure, crossover	Yes	No	No	Yes	Moderate
Scanlan et al. [12]	Case control	Yes	No	No	No	High
Nilsson et al.[9]	Prospective cohort	No	No	No	No	Very High
Cheong et al. [8]	Cross sectional	No	No	No	Yes	High
Brody et al. [15]	Randomized control trial	Yes	Yes	Yes	No	Low
Horowitz et al. [16]	Prospective cohort	No	No	No	Yes	High
Gridler et al. [14]	Randomized control trial	No	No	No	Yes	High

### Publication bias

**Fig 4:**
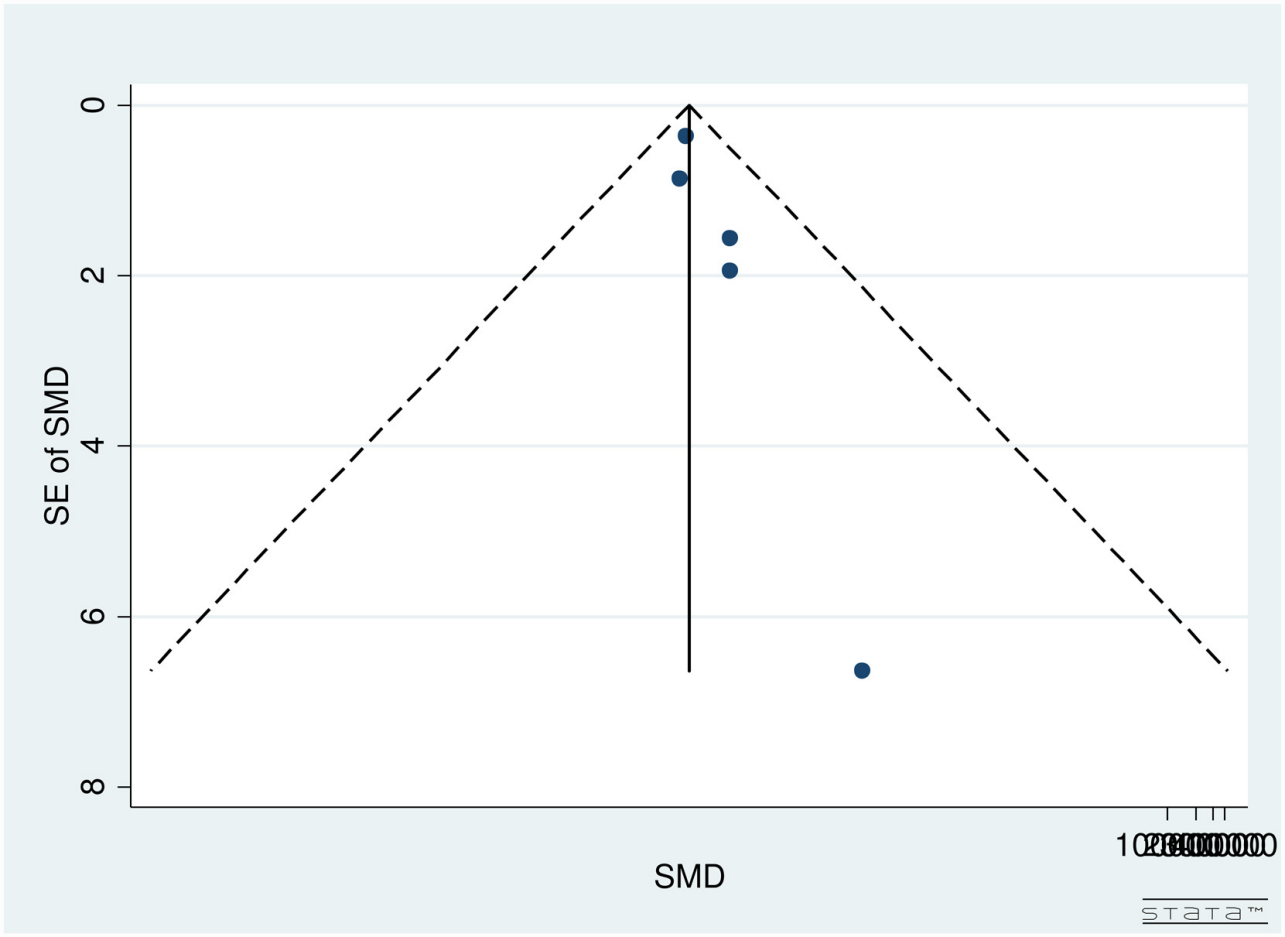
Funnel Plot for Included Studies Evaluating Reading Speed in Age Related Macular Degeneration Patients.

Publication bias for studies on reading speed was assessed through the use of a funnel plot (Fig 4). As seen in Fig 4, large studies were clustered near the top with only one smaller study at the base of the funnel plot. Therefore, due to the spread of studies in the funnel plot, publication bias could not be concluded. Partially, the reason was difficulty in interpretation of funnel plot for a small group of studies, high heterogeneity (see below) and small effect sizes. Additionally, publication bias is only one of the numerous possible explanations for funnel plot asymmetry.

**Fig 5:**
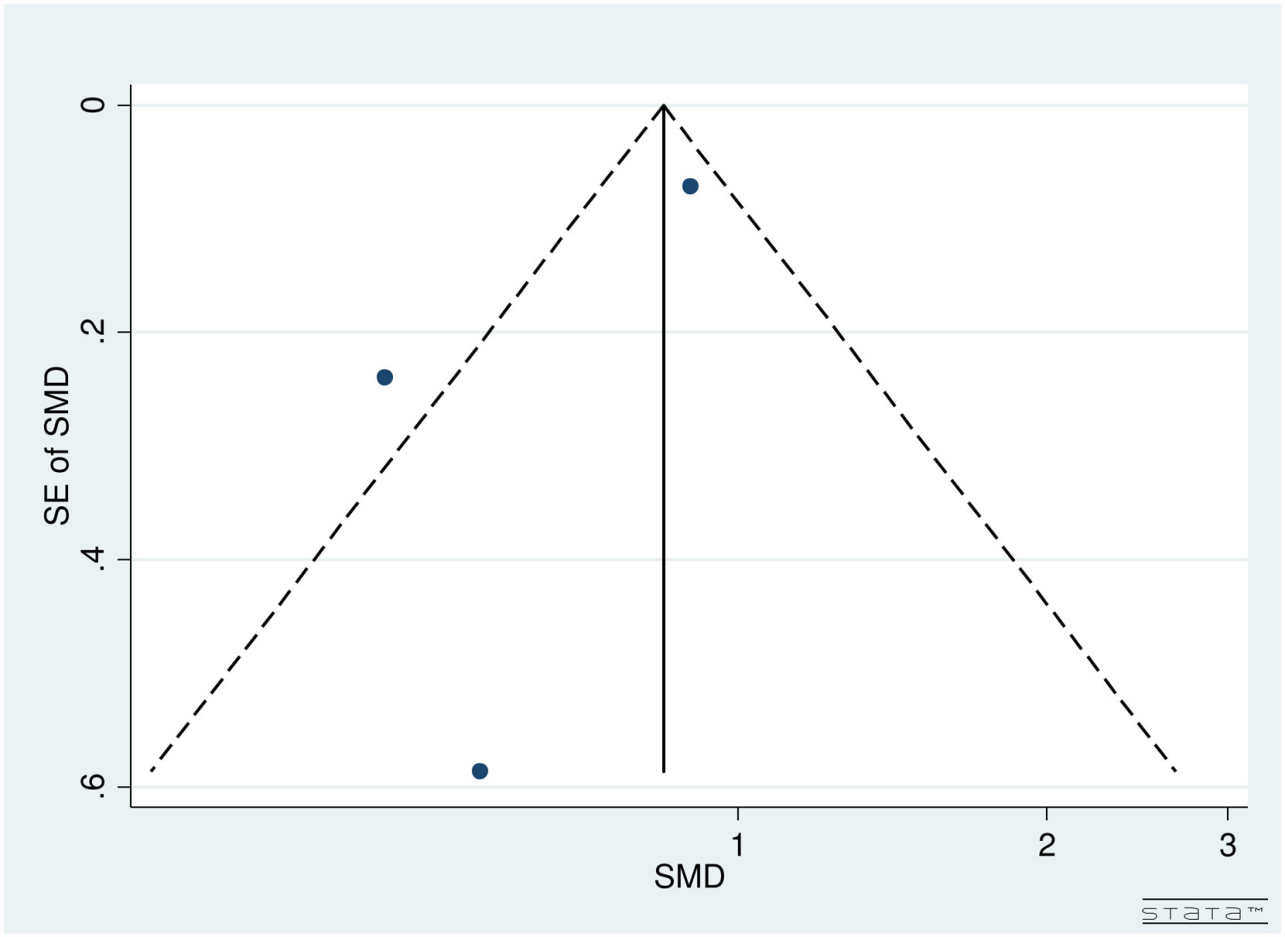
Funnel Plot for Included Studies Evaluating Depression Score in Age Related Macular Degeneration Patients.

Similarly, a funnel plot was used to assess the publication bias of the literature on depression scores. Based on the funnel plot (Fig 5), there seems to be no publication bias present. That is shown through the even spread of studies included between those that are large and those that are small.

## Discussion

This meta-analysis was conducted to compare reading speed and depression scores in patients with AMD before and after undergoing low-vision rehabilitation. Meta-analysis was done to test the effectiveness of the different low-vision rehabilitation strategies on improving reading speed and depression scores. Nine articles (885 subjects) were included from the vast area of vision rehabilitation for AMD patients indicating a necessity for research in this area.

Low-vision rehabilitation strategies including microperimetric biofeedback, microscopes teaching program and eccentric viewing training significantly improved reading speed. The maximum improvement in the reading speed was shown by eccentric viewing training program. The overall result showed a significant improvement in reading speed in those who underwent low-vision rehabilitation. The magnitude of this effect estimate of improvement is considered a small effect, which could have been a result of the heterogeneity between studies.

A number of factors likely contributed to the heterogeneity including inconsistency in differences between various low-vision rehabilitation programs, differential management, differences in the study population, potential variability in facilities to conduct these programs, differential management conducting these programs, variable follow-up periods, rates of compliance, and year the programs were conducted.

In contrast to reading speed, the improvements in depression scores were not shown to be significantly affected after rehabilitation. There is a possibility of this result of the effect estimate being highly weighted on one study because of its much larger sample size. However, based on the funnel plot of depression scores, there was no publication bias expected in the effect estimate.

There are some limitations to this study. First, all titles were accessed online and therefore, that could create some bias in terms of the inclusion of certain studies. Second, only studies published in English were included in this systematic review because of the language limitation of the authors. Despite the limitation to the English language, the studies included were from a variety of locations worldwide, therefore, any bias due to the language restriction is expected to be limited. Third, because of the inclusion criteria the number of studies eligible for the inclusion were limited. Studies with information on reading speed and depression before and after low-vision rehabilitation were included in the analysis. Further, meta-analysis of observational studies is influenced by inherent biases in the included articles [22]. For example, there could be other factors such as income status, socio-economic status, previous ocular and non-ocular surgeries, family history, other ocular and non-ocular diseases, pre-operative and post-operative medications, number of medications, comorbidities, etc. influencing the estimates in the original studies. Lastly, concrete conclusions could not be made due to smaller number of studies included in the analysis and the high risk of bias in the individual included studies. Most of the included studies had a high risk of bias mostly due to a lack in blinding and allocation concealment in those studies. Therefore, more studies with sounder methodological qualities on the subject of the effects of low vision rehabilitation strategies on reading speed and depression scores in those with AMD would go a long way in clarifying the outcomes.

It is important to note that based on this systematic review, eight papers met the inclusion criteria. Due to AMD’s large impact on individual’s psychological and economic well-being along with its high prevalence rates, such a low number of studies show a dearth in the literature. Therefore, more studies dedicated to better understand the effects of different rehabilitation strategies on reading speed and depression scores would be beneficial in furthering our understanding of the effects of low-vision rehabilitation on those with AMD.

In conclusion, low-vision rehabilitation may improve reading speed in those with AMD. However, it may not have a significant effect on depression scores in older adults with AMD. High quality research in studying the effect of low vision rehabilitation strategies and their effect on reading speed and depression scores in adults with AMD is required.

## Supporting Information

S1 File PRISMA 2009 Checklist. (DOC)

S2 File Search Strategy for MEDLINE, EMBASE, and CINAHL. (DOCX)

S3 File Level 1, 2, and 3 Screening Questions. (DOCX)
