# Supplementary material for: The Effects of Low-Vision Rehabilitation on Reading Speed and Depression in Age Related Macular Degeneration: A Meta-Analysis
Source: PLoS One. 2016 Jul 14;11(7):e0159254. doi: 10.1371/journal.pone.0159254 (PMC4945035; doi:10.1371/journal.pone.0159254)
Supplement: S2 File — (DOCX) [file pone.0159254.s002.docx]

**S2: Search Strategies**

**MEDLINE - Ovid** Jan 28,2015

| [# ▲](http://ovidsp.tx.ovid.com/sp-3.14.0b/ovidweb.cgi?&S=CALAFPLNLKDDHDGGNCLKFGLBEFOPAA00&Sort+Sets=descending) | Searches | Results |
| --- | --- | --- |
| 1 | exp Vision, Low | 2294 |
| 2 | (vision disorder* or eye disease* or vision impair* or legal* blind* or low vision).mp. [mp=title, abstract, original title, name of substance word, subject heading word, keyword heading word, protocol supplementary concept word, rare disease supplementary concept word, unique identifier] | 61449 |
| 3 | 1 or 2 | 62611 |
| 4 | exp Rehabilitation/is, ut [Instrumentation, Utilization] | 3394 |
| 5 | (low vision rehab* or home rehab* or rehab* vision impair* or low vision aid* or "aid* to low vision").mp. [mp=title, abstract, original title, name of substance word, subject heading word, keyword heading word, protocol supplementary concept word, rare disease supplementary concept word, unique identifier] | 714 |
| 6 | 4 or 5 | 4096 |
| 7 | exp Macular Degeneration/ep, et, pc, ra, rt, rh, th [Epidemiology, Etiology, Prevention & Control, Radiography, Radiotherapy, Rehabilitation, Therapy] | 5440 |
| 8 | (Retina* Disease* or Retina* Macula* Degenerat* or drusen*).mp. [mp=title, abstract, original title, name of substance word, subject heading word, keyword heading word, protocol supplementary concept word, rare disease supplementary concept word, unique identifier] | 21261 |
| 9 | 7 or 8 | 26105 |
| 10 | 3 and 6 and 9 | 79 |
| 11 | limit 10 to (english language and humans) | 62 |

**EMBASE - Ovid** Jan 28,2015

| [# ▲](http://ovidsp.tx.ovid.com/sp-3.14.0b/ovidweb.cgi?&S=NBGCFPLNJNDDHDNMNCLKCFOBJPDAAA00&Sort+Sets=descending) | Searches | Results |
| --- | --- | --- |
| 1 | visual disorder/ or exp low vision/ or visual impairment/ | 61304 |
| 2 | (legal* Blind* or vison disorder* or eye disease* or vision impair*).mp. [mp=title, abstract, subject headings, heading word, drug trade name, original title, device manufacturer, drug manufacturer, device trade name, keyword] | 48904 |
| 3 | 1 or 2 | 106047 |
| 4 | spectacles/ or reading/ or visual aid/ or low vision aid.mp. or therapy/ | 1294766 |
| 5 | (rehab* or home rehab* or rehab* vision impair* or low vision aid* or aid* to low vision).mp. [mp=title, abstract, subject headings, heading word, drug trade name, original title, device manufacturer, drug manufacturer, device trade name, keyword] | 218803 |
| 6 | 4 or 5 | 1490442 |
| 7 | retina macula age related degeneration/ or age related macular degeneration/ or retina macula degeneration/ | 20188 |
| 8 | (Retina* Disease* or Retina* Macula* Degenerat* or drusen*).mp. [mp=title, abstract, subject headings, heading word, drug trade name, original title, device manufacturer, drug manufacturer, device trade name, keyword] | 33460 |
| 9 | 7 or 8 | 33696 |
| 10 | 3 and 6 and 9 | 497 |
| 11 | limit 10 to (human and english language) | 373 |

**CINAHL** Jan 28, 2015

| [Search ID#](javascript:__doPostBack('ctl00$ctl00$FindField$FindField$historyControl$ReorderHistoryLink','')) | Search Terms | Results |
| --- | --- | --- |
| S10 | S3 AND S6 AND S9 | 47 |
| S9 | S7 OR S8 | 2,415 |
| S8 | Retina* Disease* or Retina* Macula* Degenerat* or drusen* | 1,551 |
| S7 | (MH "Macular Degeneration/EP/RH/TH") OR (MH "Eye Diseases/CO/RH/TH”) | 908 |
| S6 | S4 OR S5 | 100,686 |
| S5 | rehab* or home rehab* or rehab* vision impair* or low vision aid* or aid* to low vision | 100,620 |
| S4 | MH "Rehabilitation of Vision Impaired/ES/UT” OR MH "Eye Protective Devices/UT" | 127 |
| S3 | S1 OR S2 | 4,441 |
| S2 | legal* Blind* or vison disorder* or eye disease* or vision impair* | 4,408 |
| S1 | MH "Vision, Subnormal/RH/TH/CO/EP” | 63 |
