# Supplementary material for: The Effects of Low-Vision Rehabilitation on Reading Speed and Depression in Age Related Macular Degeneration: A Meta-Analysis
Source: PLoS One. 2016 Jul 14;11(7):e0159254. doi: 10.1371/journal.pone.0159254 (PMC4945035; doi:10.1371/journal.pone.0159254)
Supplement: S3 File — (DOCX) [file pone.0159254.s003.docx]

**S3: Screening Questions**

**Level 1 Screening:**

1) Does the study look at AMD (both wet and dry)?

1. Yes
2. No
3. Unclear

2) Is the study looking at low vision rehabilitation strategies?

1. Yes
2. No
3. Unclear

**Level 2 Screening:**

1. Is it a research study (not an editorial, opinion, case report)?
2. Yes
3. No
4. Unclear

**Level 3 Screening:**

1. Does the study look at low vision specifically (20/60 or worse)?
   - 1. Yes
     2. No
     3. Unclear
2. Does the study look at adults aged 55 and older?
   - 1. Yes
     2. No
     3. Unclear
3. Is the sample size 20 eyes or greater?
   - 1. Yes
     2. No
     3. Unclear
